# Cardiac autonomic dysfunction in children and adolescents with obesity: a systematic review and meta-analysis

**DOI:** 10.1590/1984-0462/2026/44/2026017

**Published:** 2026-09-18

**Authors:** Thales da Fonseca Dornelles, Júlia Lima Barrozo

**Affiliations:** a Faculdade de Ciências Médicas e da Saúde, Juiz de Fora, MG, Brasil.

**Keywords:** Pediatric obesity, Heart rate variability, Autonomic nervous system, Systematic review, Meta-analysis, Obesidade pediátrica, Variabilidade da frequência cardíaca, Sistema nervoso autônomo, Revisão sistemática, Metanálise

## Abstract

**Objective:**

To evaluate the association between excess weight and cardiac autonomic modulation in children and adolescents.

**Data source:**

PubMed, Embase, and the Cochrane Central Register of Controlled Trials were searched from inception to April 2, 2026 for observational studies comparing short-term resting heart rate variability between children and adolescents with excess weight and normal-weight controls. The protocol was registered in PROSPERO (CRD420251005501).

**Data synthesis:**

Eighteen studies comprising 1,590 participants were included. Random-effects meta-analyses using standardized mean differences showed lower root mean square of successive differences of RR intervals (RMSSD −1.00; 95% confidence interval [95%CI] −1.68 to −0.31), lower percentage of successive normal-to-normal intervals differing by more than 50 ms (pNN50 −0.48; 95%CI −0.68 to −0.28), and higher low-frequency/high-frequency ratio (LF/HF 0.70; 95%CI 0.30 to 1.10) in the excess weight group. No significant differences were observed for the standard deviation or mean of normal-to-normal intervals. Leave-one-out analyses supported directional robustness, and between-group body mass index difference significantly moderated LF/HF (β=-0.15; p=0.045), but not RMSSD.

**Conclusions:**

Pediatric excess weight appears to be associated with impaired cardiac autonomic modulation, mainly characterized by lower parasympathetic-related heart rate variability indices. Among the evaluated parameters, pNN50 showed the most consistent reduction, whereas RMSSD showed the largest pooled effect.

## RESUMO

**Objetivo:**

Avaliar a associação entre sobrepeso/obesidade e modulação autonômica cardíaca em crianças e adolescentes.

**Fontes de dados:**

PubMed, Embase e Cochrane Central Register of Controlled Trials foram pesquisadas até 2 de abril de 2026 para estudos observacionais que compararam a variabilidade da frequência cardíaca de curta duração, em repouso, entre crianças e adolescentes com sobrepeso/obesidade e controles eutróficos. O protocolo da revisão sistemática foi registrado no PROSPERO (CRD420251005501).

**Síntese dos dados:**

Dezoito estudos, com 1.590 participantes, foram incluídos. Metanálises de efeitos aleatórios com diferença média padronizada mostraram menores valores de raiz quadrada da média quadrática das diferenças sucessivas entre intervalos RR (RMSSD −1,00; intervalo de confiança de 95% [IC95%] −1,68 a −0,31), menores valores da porcentagem de diferenças superiores a 50 ms entre pares de intervalos de batimentos cardíacos normais consecutivos (pNN50 −0,48; IC95% −0,68 a −0,28) e maior relação de baixa frequência/alta frequência (LF/HF 0,70; IC95% 0,30 a 1,10) no grupo com sobrepeso/obesidade. Não houve diferenças significativas para o desvio-padrão ou para a média dos intervalos normais entre batimentos cardíacos consecutivos. As análises de sensibilidade por omissão de um estudo por vez (leave-one-out) sustentaram a robustez direcional dos resultados e a diferença no índice de massa corporal entre os grupos moderou significativamente LF/HF (β=-0.15; p=0.045), mas não RMSSD.

**Conclusões:**

O excesso de peso pediátrico parece estar associado à modulação autonômica cardíaca prejudicada, principalmente refletida por menores índices vagais de variabilidade da frequência cardíaca. Entre os parâmetros avaliados, o pNN50 mostrou a redução mais consistente, enquanto o RMSSD apresentou o maior efeito combinado.

## INTRODUCTION

Obesity is a major global public health concern, and its cardiovascular and metabolic consequences are increasingly recognized from childhood onward.^1^ Among these consequences, cardiac autonomic dysfunction, characterized by an imbalance between the sympathetic and parasympathetic branches of the autonomic nervous system, has emerged as a potential early marker of cardiovascular risk in pediatric populations.^2^

Heart rate variability (HRV) is a widely validated, non-invasive marker of cardiac autonomic regulation, and reduced HRV has been consistently associated with increased risk of arrhythmias, cardiovascular events, and all-cause mortality in adulthood.^3,4^ During childhood and adolescence, a predominance of parasympathetic activity contributes to a cardioprotective autonomic profile.^5^ Excess adiposity during this developmental period may disrupt the maturation of neurocardiovascular control mechanisms, with potential long-term health implications.^6^

Despite the clinical relevance of this topic, the evidence on the association between excess weight and HRV in children and adolescents remains inconsistent. While some studies report significant reductions in vagally mediated HRV indices in pediatric populations with excess adiposity,^2,7^ others have found no statistically significant differences compared with normal-weight peers.^8,9^ This inconsistency may reflect not only biological variability - for example, variations in pubertal stage, cardiorespiratory fitness, duration of obesity exposure, and fat distribution - but also methodological heterogeneity across studies, including differences in HRV metrics, recording conditions, and statistical approaches. To date, no systematic synthesis has integrated these findings while explicitly accounting for this heterogeneity and exploring how the magnitude of adiposity contrast between groups may influence pooled estimates.

To address this gap, a systematic review and meta-analysis was conducted to evaluate the association between excess weight and cardiac autonomic modulation, assessed through HRV indices, in children and adolescents.

## METHOD

This review was conducted in accordance with the Preferred Reporting Items for Systematic Reviews and Meta-Analyses (PRISMA) guidelines.^10^ The study protocol was registered with the International Prospective Register of Systematic Reviews (PROSPERO; National Institute for Health and Care Research, York, United Kingdom) under registration CRD420251005501.

Eligible studies were observational (cross-sectional, cohort, or case–control) and investigated the association between excess weight and autonomic function using short-term HRV recordings obtained under standardized awake conditions. Baseline data from clinical trials were also included when they provided cross-sectional comparisons between groups prior to any intervention. Short-term rather than 24-hour ambulatory HRV recordings were selected because they allow data collection under more controlled conditions. This reduces methodological variability related to circadian influences, physical activity, and uncontrolled environmental factors.^11^

Studies were excluded if they:

1. Did not include a comparison between individuals with excess weight and a normal-weight control group;
2. Assessed autonomic function using methods other than HRV or did not report short-term HRV measured at rest under standardized awake conditions;
3. Included participants with conditions known to significantly affect autonomic function (e.g., cardiovascular, neurological, or endocrine disorders) without appropriate adjustment or stratification;
4. Involved interventions, acute experimental conditions, or post-exercise measurements without reporting baseline data;
5. Were conducted in adult populations or did not provide separate data for children and adolescents;
6. Lacked sufficient data for extraction or effect size calculation; or
7. Were conference abstracts, reviews, case reports, editorials, or non-original studies.

The review question was defined according to the PECO framework (Population, Exposure, Comparison, Outcome): "Do overweight or obese children and adolescents exhibit altered cardiac autonomic modulation compared with their normal-weight peers?". In this approach, (P) represents children and adolescents aged 5–18 years, of both sexes; (E) refers to overweight or obesity (excess weight) diagnosed using objective and clearly described criteria; (C) consists of a control group composed of normal-weight children and adolescents; and (O) comprises quantitative measures of cardiac autonomic function assessed by HRV. These measures include root mean square of successive differences of RR intervals (RMSSD), percentage of successive normal-to-normal intervals differing by more than 50 ms (pNN50), standard deviation of normal-to-normal intervals (SDNN), low-frequency/high-frequency ratio (LF/HF), and mean normal-to-normal intervals (MNN).

A systematic search of published studies was conducted in the databases PubMed, Embase, and the Cochrane Central Register of Controlled Trials (CENTRAL). The searches were conducted through April 2, 2026. For each database, controlled vocabulary terms (e.g., Medical Subject Headings [MeSH] and Emtree) and free-text terms were combined and adapted to the specific syntax and search fields of each platform. Boolean operators ("AND" and "OR") were used to combine search terms.

The study selection process was conducted independently by two reviewers. Duplicate records were removed through automated and manual procedures within the Rayyan systematic review management platform. Titles and abstracts were screened independently by the same two reviewers. Potentially eligible studies were subsequently assessed through full-text review. Any disagreements were resolved through discussion and consensus.

Data extraction was performed independently by two reviewers using a standardized data extraction form in Microsoft Excel. Extracted data included first author, year of publication, study design, participant characteristics (e.g., age, sex, and sample size), and HRV parameters. Discrepancies were resolved by discussion and consensus.

The methodological quality and risk of bias of the included studies were independently assessed by two authors using the Joanna Briggs Institute (JBI) critical appraisal tools for analytical observational studies. The tool considers criteria related to sample selection, exposure and outcome measurement, and control of confounding factors. Furthermore, the quality of evidence and strength of recommendations were evaluated using the Grading of Recommendations Assessment, Development, and Evaluation (GRADE) approach.

Effect sizes for the predefined HRV outcomes were calculated as standardized mean differences (SMDs; Hedges’ g) with corresponding sampling variances, based on reported means, standard deviations, and sample sizes in the obese/overweight (OB/OW) and normal-weight (NW) groups. Pooled estimates were obtained using random-effects models with restricted maximum likelihood (REML) estimation.

Statistical heterogeneity was assessed using the I^2^ statistic and interpreted according to the approximate thresholds proposed by Higgins et al. (0–40%: might not be important; 30–60%: may represent moderate heterogeneity; 50–90%: may represent substantial heterogeneity; 75–100%: considerable heterogeneity), while also considering the magnitude and direction of effects and the clinical and methodological diversity across studies.^12^

To investigate whether the magnitude of body mass index (BMI) difference between groups (ΔBMI) contributed to heterogeneity, a random-effects meta-regression was conducted using ΔBMI as a continuous moderator. Results were summarized using the regression coefficient (β), 95% confidence interval (CI), p-value, omnibus moderator test (QM), residual τ^2^, and the R^2^ analog, defined as the proportional reduction in between-study variance relative to the intercept-only random-effects model.

Robustness of the pooled estimate was examined through leave-one-out sensitivity analysis, in which each study was sequentially removed, and the model was re-estimated. Potential small-study effects were assessed by visual inspection of funnel plot asymmetry and formally tested using Egger's regression test. All analyses were performed in R using the metafor package.

## RESULTS

**Figure 1:**
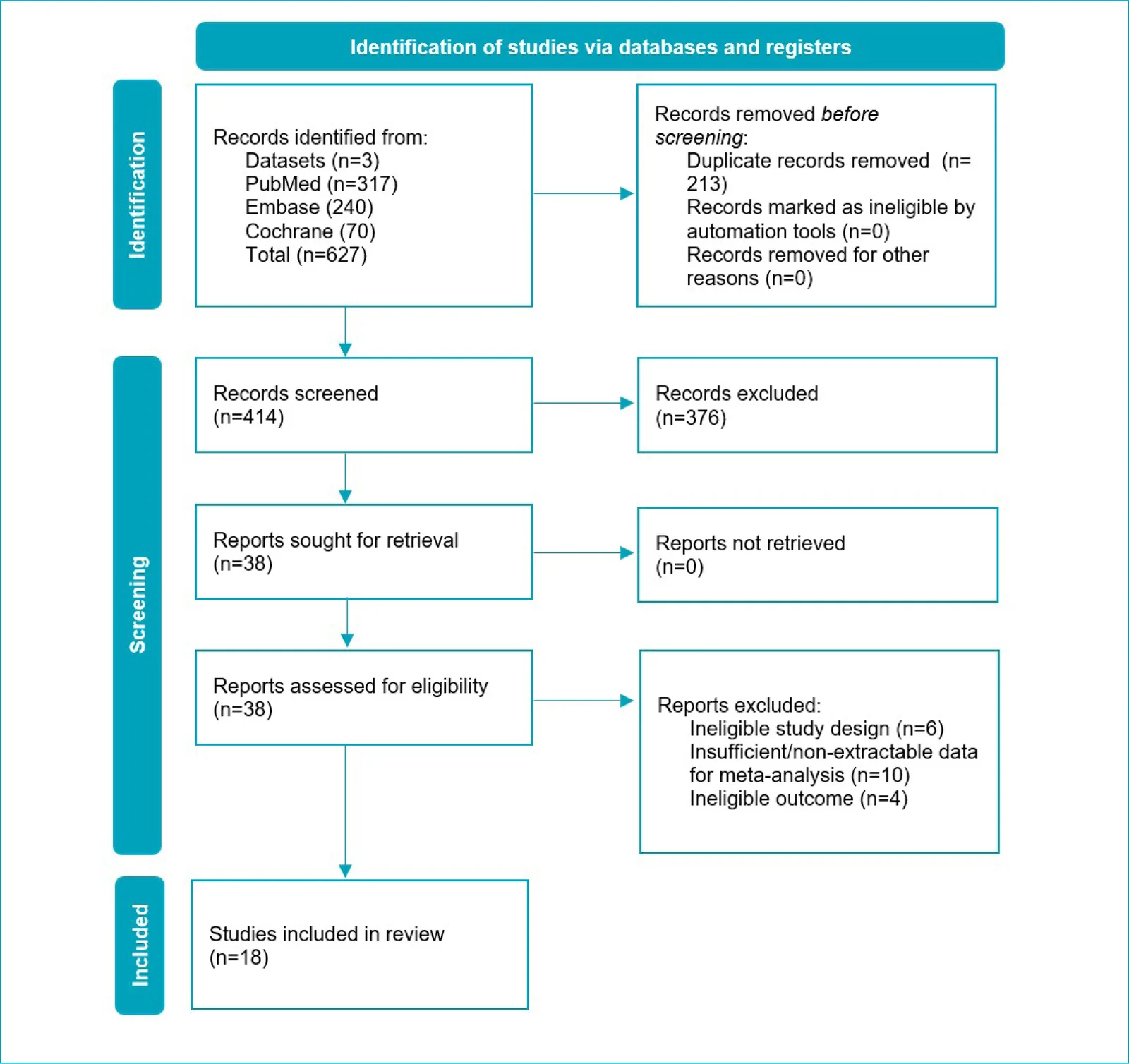
Preferred reporting items for systematic reviews and meta-analyses flow diagram.

Initially, 627 studies were identified across the selected databases as potentially relevant to the research objectives (Figure 1). After removing duplicate records and ineligible studies, 414 remained.

Score: Prepared by the authors.

In the second screening phase, 38 records underwent full-text review. Of these, 18 met all eligibility criteria and were included in the quantitative synthesis, whereas 20 were excluded based on the predefined criteria. Overall, the 18 studies included 1,590 children and adolescents.

These studies used different observational designs and evaluated basal autonomic modulation in children and adolescents with OB/OW compared with NW controls. Not all studies reported all HRV outcomes; therefore, the number of studies contributing to each pooled analysis varied by parameter, ranging from 7 for pNN50 to 15 for LF/HF. The main descriptive and methodological characteristics of the included studies are summarized in Table 1 ^13–27^.

**Table 1:** Characteristics of the studies included in the meta-analysis of heart rate variability in children and adolescents with excess weight compared with normal-weight peers.

Study	Country	n	Age (y)	Obesity criteria	Recording protocol	HRV domains
Neves et al.^13^	Brazil	124	9.3±1.2	BMI Z score (WHO)	Polar H10 + Kubios; supine; 10/5 min	Time; frequency; nonlinear
Freitas et al.^2^	Brazil	66	12±3	BMI percentile (≥95th)	Polar S810i; supine; 10/5 min	Time; frequency
Campos et al.^14^	Brazil	110	~9	BMI-for-age (WHO)	5-lead ECG; supine; 15/5 min	Time; frequency; nonlinear
Reck et al.^8^	Brazil	52	13±1	BMI (pediatric)	Polar/Kubios; supine; 10/5 min	Time; frequency
Santos-Magalhães et al.^15^	Portugal	50	8.3±1	BMI percentile	Polar/Kubios; supine; 15/5 min	Time; frequency
Farinatti et al.^16^	Brazil	44	13–17	BMI Z score	Finometer; supine; ~15/5 min	Time; frequency
Mazurak et al.^17^	Germany	87	13±2	BMI-SDS (Z score)	ECG; baseline rest; ~20/5 min	Time; frequency
Paschoal et al.^18^	Brazil	30	10±1	BMI percentile (≥95th)	Polar; supine; 12/5 min	Time; frequency
Calcaterra et al.^9^	Italy	114	13±2	BMI Z score (WHO)	ECG + BP; supine; ~10 min	Time; frequency; baroreflex
Vanderlei et al.^19^	Brazil	121	10±1	BMI (pediatric)	Polar S810i + Kubios; supine; 20/~5 min	Time; frequency; nonlinear
Guízar et al.^20^	Mexico	70	14±1	BMI percentile (>95th)	Holter ECG; resting; ~60 min	Time; frequency
Kaufman et al.^21^	USA	36	11±1	BMI percentile	3-lead ECG; supine; 15/5 min	Time; frequency; nonlinear
Latchman et al.^22^	USA	37	9.0±1.5	BMI percentile (≥95th)	ECG + BP; paced breathing; 5/5 min	Frequency; baroreflex
Birch et al.^23^	UK	182	9.1±1.4	BMI (IOTF)	Suunto/Kubios; supine; 15/5 min	Time; frequency
Dangardt et al.^24^	Sweden	311	8–16	BMI Z score	ECG + BP; supine; ~20/≥5 min	Time; baroreflex
Sekine et al.^25^	Japan	16	9.2±0.3	BMI (pediatric)	Holter ECG; supine; 10/5 min	Time; frequency
Magalhães et al.^26^	Brazil	212	15.6±1.9	BMI Z score (WHO)	12-lead ECG; supine; 10/≥5 min	Time; frequency
Christaki et al.^27^	Greece	121	9±2	BMI Z score	PPG; seated, fasted; 5/5 min	Time; frequency

BMI: body mass index; BP: blood pressure; ECG: electrocardiogram; HRV: heart rate variability; N: total sample size; IOTF: International Obesity Task Force; PPG: photoplethysmography; SDS: standard deviation score; UK: the United Kingdom; USA: the United States of America; WHO: World Health Organization.

HRV domains were classified from the parameters reported by each study. Recording protocol is reported as device/software; body position or recording condition; total recording/analyzed duration, when available.

Score: Prepared by the authors.

**Figure 2:**
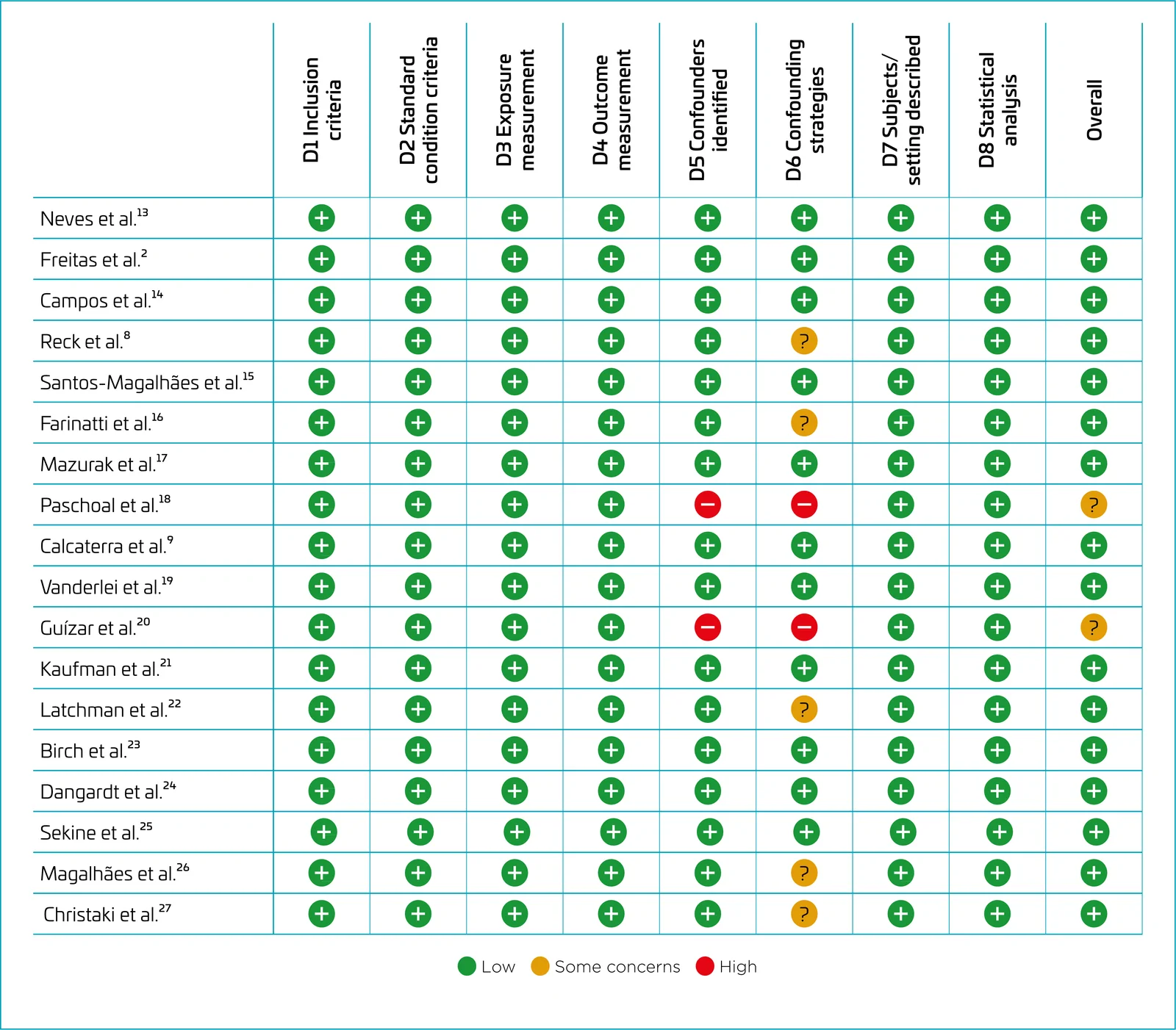
Risk of bias assessment of the included studies using design-specific Joanna Briggs Institute critical appraisal tools.

Risk of bias was assessed using design-specific JBI critical appraisal tools. Given the inclusion of cross-sectional, case-control, and retrospective cohort studies, each study was appraised using the corresponding checklist. For synthesis and visualization purposes, common methodological domains across tools were harmonized and summarized in Figure 2. Overall, most studies met methodological quality criteria, with limitations observed mainly in domains related to the identification and control of potential confounding factors, a common challenge in pediatric observational research.

Score: Prepared by the authors.

Funnel plots were generated for all five HRV outcomes to visually assess potential small-study effects. Visual inspection did not reveal strong or consistent asymmetry across outcomes. Some dispersion was observed for LF/HF, SDNN, and MNN; however, these patterns were difficult to distinguish from the high between-study heterogeneity and the limited number of studies available for some outcomes, particularly pNN50. Overall, funnel plot inspection did not suggest clear evidence of major small-study effects.

To statistically assess funnel plot asymmetry, Egger's regression test was performed for each outcome using mixed-effects meta-regression models with standard error as the predictor. Egger's regression test did not show statistically significant asymmetry for RMSSD (p=0.817), LF/HF (p=0.124), SDNN (p=0.942), or MNN (p=0.364). For pNN50, the result was borderline (p=0.072) and should be interpreted cautiously, given the small number of studies. Overall, these analyses did not provide strong evidence of funnel plot asymmetry or major small-study effects.

**Figure 3:**
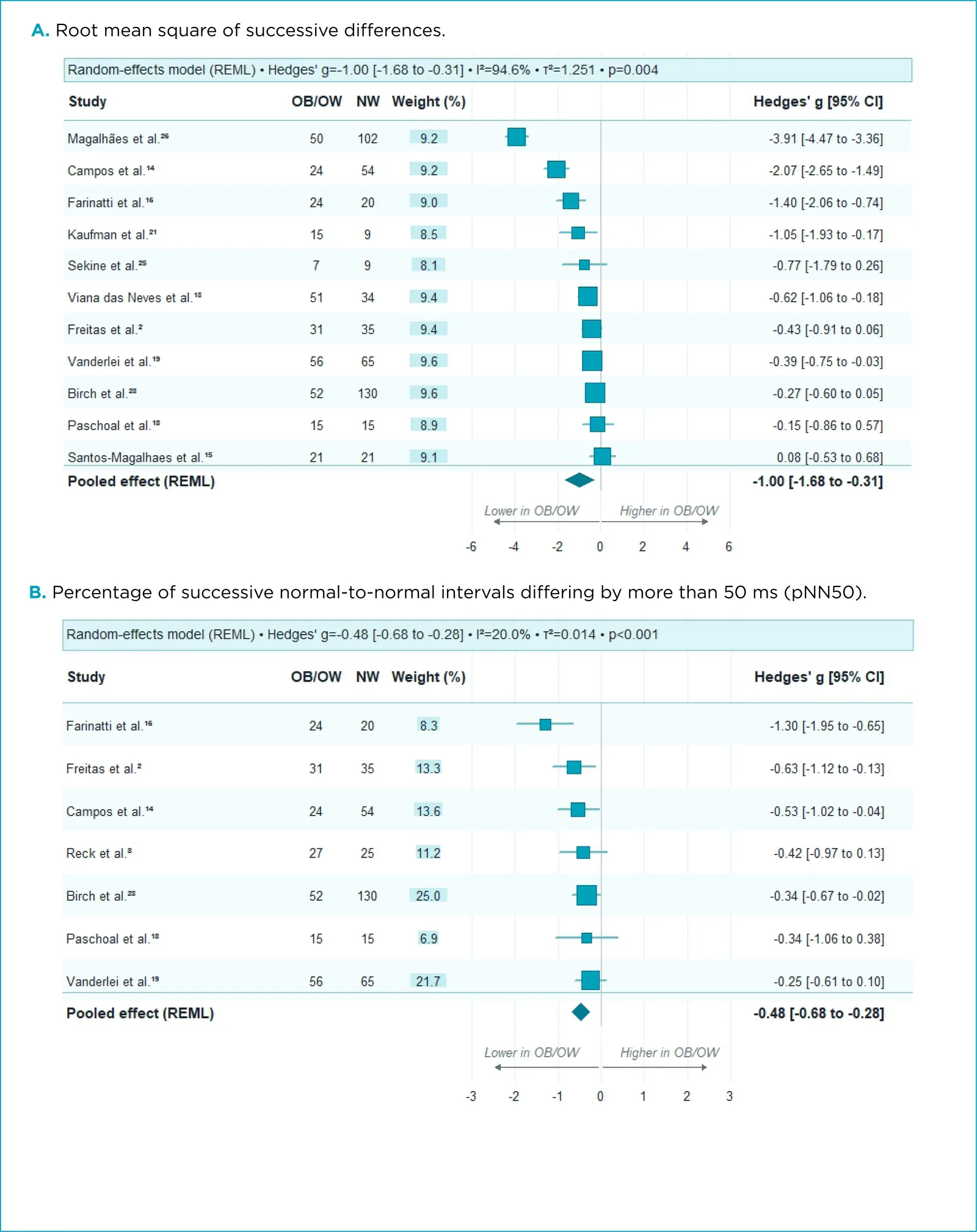

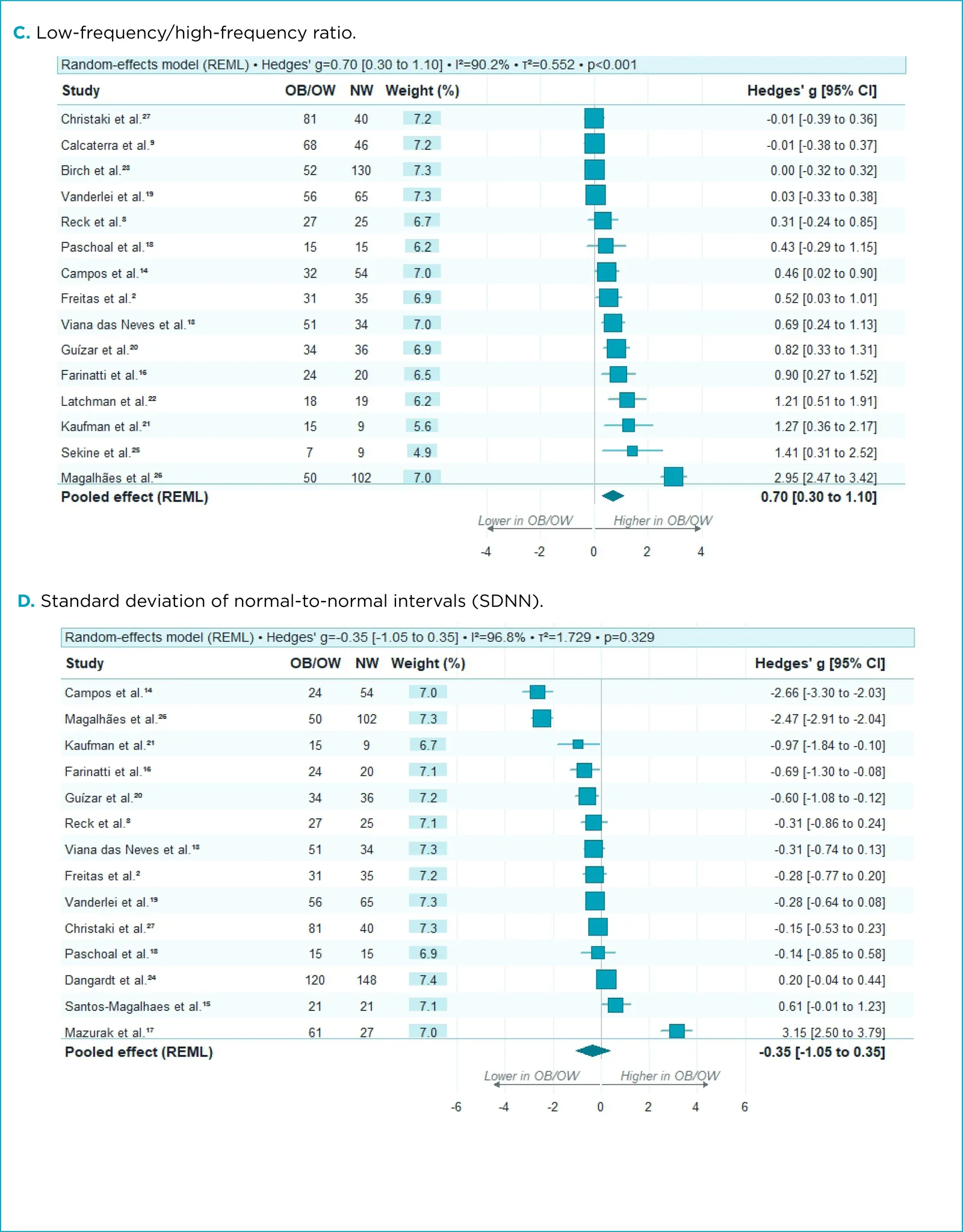

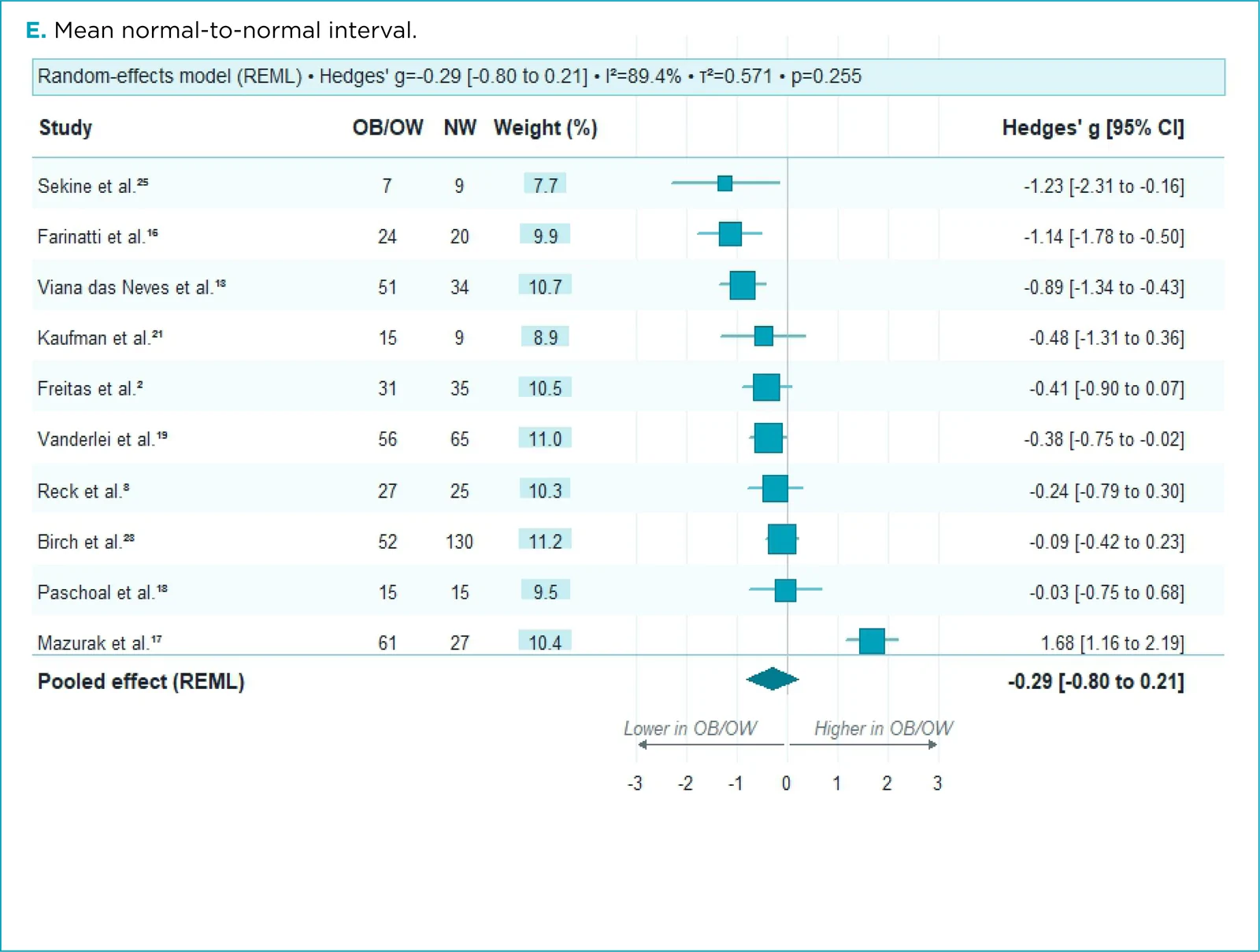
Forest plots of pooled heart rate variability differences between obese/overweight children and adolescents and normal-weight controls: (A) RMSSD, (B) pNN50, (C) LF/HF, (D) SDNN, and (E) MNN.

Across HRV parameters, excess weight in pediatric populations was associated with significant alterations in selected autonomic indices, although the strength and consistency of these associations varied across outcomes. Forest plots for the pooled analyses of RMSSD, pNN50, LF/HF, SDNN, and MNN are shown in Figure 3.

Score: Prepared by the authors.

REML: Random-effects model; OB: obese; OW: overweight; NW: normal-weight; CI: confidence interval.

OB/OW: obese/overweight; NW: normal weight; RMSSD: root mean square of successive differences; pNN50: percentage of successive normal-to-normal intervals differing by more than 50 ms; LF/HF: low-frequency/high-frequency ratio; SDNN: standard deviation of normal-to-normal intervals; MNN: mean normal-to-normal interval.

For RMSSD, 11 studies comprising 840 participants were included. The pooled effect showed significantly lower RMSSD values in the OB/OW group than in NW controls (SMD −1.00; 95%CI −1.68 to −0.31; p=0.0045), with considerable heterogeneity (I^2^=94.6%). For pNN50, seven studies including 573 participants were analyzed, showing a significant reduction in the OB/OW group (SMD −0.48; 95%CI −0.68 to −0.28; p<0.001) with low heterogeneity (I^2^=20.0%). LF/HF ratio was assessed in 15 studies totaling 1192 participants and was significantly higher in the OB/OW group (SMD 0.70; 95%CI 0.30 to 1.10; p<0.001), with high heterogeneity (I^2^=90.1%).

For SDNN, 14 studies with 1241 participants yielded a non-significant pooled effect (SMD −0.35; 95%CI −1.05 to 0.35; p=0.33; I^2^=96.8%). For MNN, 10 studies including 708 participants also showed no significant difference (SMD −0.29; 95%CI −0.80 to 0.21; p=0.26; I^2^=89.4%). Overall, the pooled results indicate lower RMSSD and pNN50 values and higher LF/HF ratios in children and adolescents with excess weight, whereas SDNN and MNN remained inconclusive.

A leave-one-out sensitivity analyses for all outcomes were performed. Overall, the omission of individual studies did not materially change the direction of the pooled estimates, supporting the robustness of the main findings. For pNN50, the pooled effect remained stable and heterogeneity stayed low to moderate across iterations, indicating no disproportionate influence of any single study. For RMSSD and LF/HF, however, Magalhães et al.,^26^ 2020 emerged as the main contributor to between-study heterogeneity: its exclusion reduced I^2^ from 94.6 to 82.4% for RMSSD and from 90.1 to 64.3% for LF/HF, although the pooled effects remained in the same direction. In contrast, no single study substantially altered the heterogeneity pattern for SDNN, with I^2^ remaining very high throughout the leave-one-out analyses, despite a modest decrease after omitting Mazurak et al.,^17^ 2016. For MNN, most leave-one-out estimates remained close to the overall estimate, whereas exclusion of Mazurak et al.,^17^ 2016 produced a marked reduction in heterogeneity, from 89.4 to 51.7%. Taken together, these analyses indicate that the pooled results were broadly robust to single-study omission, although specific studies contributed disproportionately to heterogeneity in RMSSD, LF/HF, and MNN.

**Figure 4:**
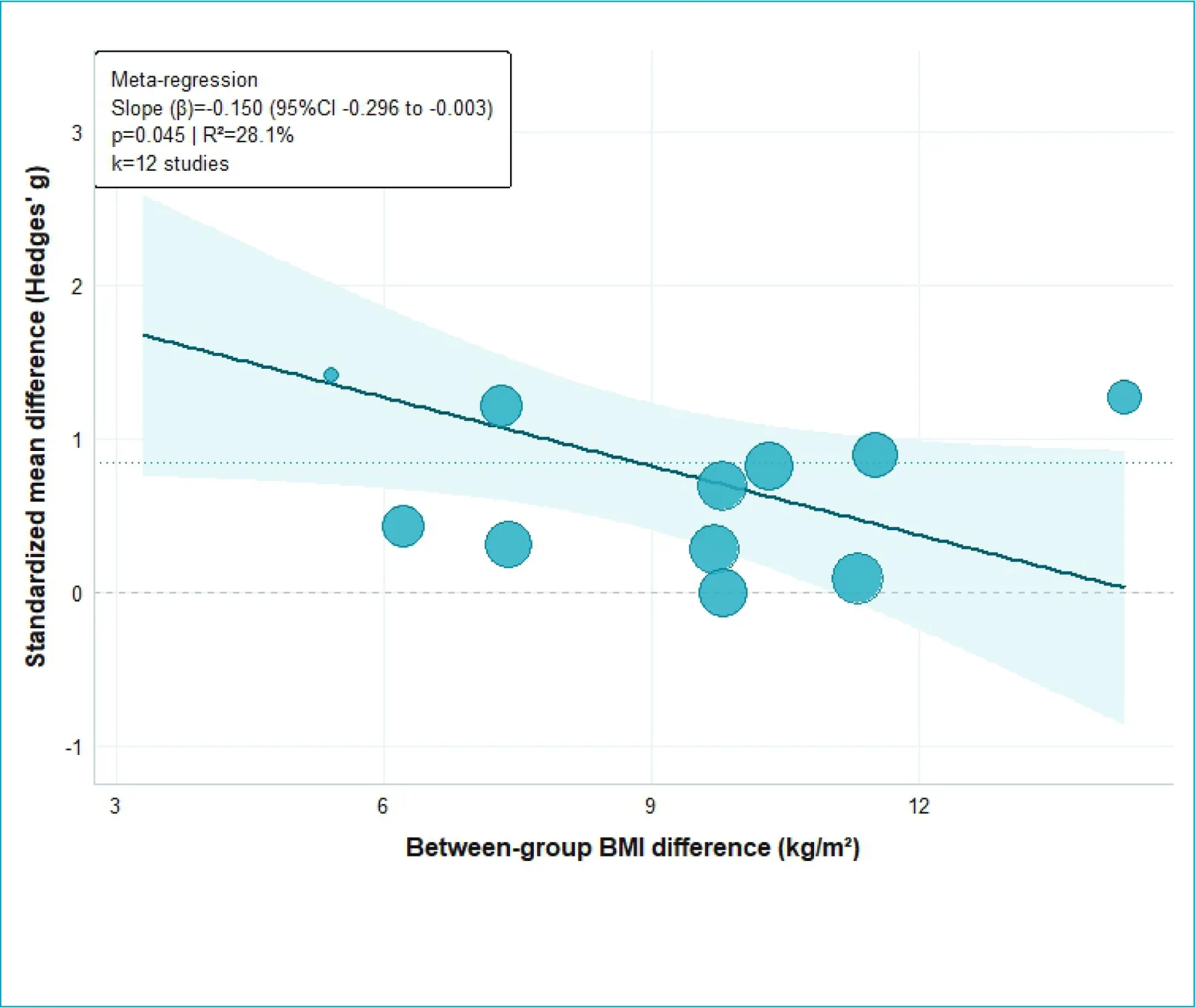
Meta-regression bubble plot of low-frequency/high-frequency ratio effect size on between-group body mass index difference.

BMI: body mass index; CI: confidence interval.

For the LF/HF meta-regression, 12 of the 15 studies were included, as three did not provide sufficient data to calculate between-group BMI difference. The model showed a significant association between BMI difference and LF/HF effect size (β=-0.15; p=0.045), explaining 28.1% of the between-study heterogeneity (Figure 4). For RMSSD, seven studies were included, and no significant association was observed (β=0.06; p=0.620).

Score: Prepared by the authors.

Using the GRADE approach, the certainty of evidence across HRV outcomes ranged from low to very low. pNN50 was supported by low-certainty evidence, whereas RMSSD, LF/HF, SDNN, and MNN were rated as having very low-certainty evidence. These ratings suggest that, while several pooled estimates point toward altered autonomic modulation in pediatric excess weight, the certainty of the evidence supporting these associations remains limited by its observational nature and, for most outcomes, is primarily downgraded due to inconsistency.

## DISCUSSION

The present meta-analysis supports the presence of altered cardiac autonomic modulation in children and adolescents with excess weight but also shows that these alterations are not captured uniformly across HRV metrics. The clearest pooled abnormalities were observed for RMSSD, pNN50, and LF/HF, whereas SDNN and MNN did not show significant between-group differences. Importantly, the degree of heterogeneity varied markedly across outcomes, suggesting that both physiological specificity and methodological variability may influence these findings.

In this context, factors such as duration and severity of obesity exposure, pubertal stage, and cardiorespiratory fitness may modulate early autonomic manifestations and hinder their detection, particularly when less specific HRV parameters are used.^28,29^ Supporting this possibility, Gutin et al.^30^ demonstrated in 304 adolescents that more favorable HRV profiles (higher RMSSD, lower LF in normalized units) were independently associated with greater moderate-to-vigorous physical activity, better cardiorespiratory fitness, and lower visceral and subcutaneous adiposity, with interaction effects involving race and sex. Respiratory patterns may represent another important source of variability, as they strongly influence vagally mediated HRV indices and are themselves altered in pediatric obesity through higher resting respiratory frequency, reduced tidal volume, and a higher prevalence of sleep-disordered breathing.^31–33^ Together, these findings highlight that adiposity-related autonomic modulation in youth is not uniform and is shaped by multiple concurrent factors that may attenuate or obscure between-group differences in pooled estimates.

Among the analyzed indices, pNN50 provided the most internally consistent signal, showing a significant reduction with low heterogeneity. RMSSD was also significantly reduced and showed the largest pooled effect, supporting impaired short-term vagally mediated cardiac modulation in pediatric excess weight. However, the very high heterogeneity observed for RMSSD indicates substantial variability in effect magnitude across studies.

The mechanisms underlying these autonomic alterations in pediatric excess weight are likely multifactorial and involve metabolic, inflammatory, and adiposity-related pathways acting in concert. In adolescents with obesity, hyperglycemia and systemic low-grade inflammation have been identified as primary determinants of reduced vagally mediated HRV and altered frequency-domain indices, even across the spectrum of glycemic regulation.^34^ Within this framework, excess adiposity - particularly visceral adipose tissue - has been consistently associated with reduced vagally mediated HRV indices and increased sympathovagal imbalance in youth, suggesting a depot-specific contribution of central fat to cardiac autonomic dysregulation.^35^ Longitudinal evidence further supports a dose-dependent relationship, as higher BMI and central adiposity predict progressive deterioration of HRV indices over time in pediatric populations.^36^ Collectively, these findings suggest that the parasympathetic impairment captured by short-term HRV indices in the present meta-analysis may reflect an early manifestation of a broader cardiometabolic phenotype in which adiposity, dysglycemia, and subclinical inflammation converge to disrupt autonomic regulation from early life.

Part of this mechanistic evidence derives from pediatric populations with more advanced metabolic compromise, such as type 1 diabetes or dysglycemia. Nonetheless, the same underlying pathways - including insulin resistance, adipokine dysregulation, and low-grade inflammation - are also detectable in attenuated form in children and adolescents with uncomplicated obesity, supporting a qualitatively similar, though quantitatively milder, mechanistic profile across the broader pediatric excess weight spectrum.^37^

LF/HF ratio was likewise significantly elevated in the excess weight group, but this result warrants cautious interpretation. Although LF/HF has traditionally been used as an index of sympathovagal balance, its physiological meaning is neither singular nor stable across contexts, particularly in pediatric populations. This has been extensively demonstrated in the literature. Goldstein et al.,^38^ for example, showed that individuals with clearly elevated sympathetic tone, such as patients with congestive heart failure, may paradoxically exhibit reduced LF power. Similarly, pharmacological sympathetic stimulation with isoproterenol has been associated with LF reduction rather than elevation. More broadly, Billman's critical appraisal highlighted that LF power reflects a composite of sympathetic, parasympathetic, and baroreflex contributions rather than sympathetic activity alone, which fundamentally undermines the LF/HF ratio as a direct index of sympathovagal balance.^39^ Accordingly, the higher LF/HF ratio observed in this meta-analysis is more appropriately interpreted as evidence of altered autonomic regulation than as a direct marker of sympathetic predominance.

By contrast, SDNN and MNN did not demonstrate significant pooled effects and were both characterized by marked heterogeneity - findings that are physiologically plausible and methodologically informative. SDNN reflects overall variability across the recording period and incorporates multiple influences beyond short-term vagal modulation.^31^ This composite nature likely reduces its sensitivity to subtle obesity-related autonomic changes under resting or non-standardized conditions. Notably, although RMSSD also showed very high heterogeneity across studies, it yielded a significant pooled effect, a divergence that likely reflects the greater physiological specificity of RMSSD for short-term vagal modulation, which preserves its capacity to detect obesity-related autonomic alterations even under non-uniform recording conditions.

MNN was included because it is consistently reported in pediatric HRV studies and reflects the average cardiac chronotropic state, which is itself sensitive to autonomic tone through the non-linear inverse relationship between RR interval and heart rate.^40^ However, because MNN represents mean RR interval duration rather than beat-to-beat variability itself, it is expected to be less responsive to subtle alterations in autonomic modulation, and its null pooled result is consistent with this theoretical limitation.

Taken together, the absence of significant pooled differences for SDNN and MNN is not evidence against autonomic impairment in pediatric excess weight; rather, it suggests that broader or less specific HRV measures are less informative than short-term vagally mediated indices for detecting early autonomic alterations in this population and may require longer recording durations or standardized provocation protocols to yield interpretable signals.

The meta-regression findings further suggest that adiposity contrast between groups may affect HRV outcomes differently depending on the parameter examined. Between-group BMI difference significantly moderated LF/HF effect sizes, with larger BMI contrasts associated with smaller LF/HF effects. Although apparently counterintuitive, this pattern may reflect a context-dependent and potentially non-linear relationship between adiposity and autonomic regulation rather than a simple dose-response model. In this regard, the study by Grandemange et al.^41^ is particularly relevant as it showed that adiposity-related autonomic alterations were more evident under tachycardic or stress-like conditions than during resting recordings, were sex-dependent, and were also observed outside the highest adiposity range. These observations offer a plausible explanation for why larger between-group BMI differences may not translate into proportionally greater resting LF/HF differences. In contrast, BMI difference did not significantly moderate RMSSD effect sizes, suggesting that between-study variation in adiposity contrast did not meaningfully account for RMSSD heterogeneity, which aligns with previous findings suggesting that waist-to-height ratio and regional fat distribution may be more sensitive markers of pediatric HRV alterations than BMI itself.^42^

These findings carry important implications for future research design. Selecting samples with larger BMI contrasts does not necessarily increase sensitivity for detecting autonomic alterations, particularly for frequency-domain indices. Rather, future studies should adopt standardized recording protocols - including controlled respiratory patterns, defined body position, and harmonized recording durations - and should systematically report potential modifiers such as pubertal stage, cardiorespiratory fitness, and duration of obesity exposure. Incorporating regional adiposity measures and, where feasible, autonomic provocation protocols may further improve the detectability and interpretability of obesity-related autonomic impairment. Additionally, longitudinal studies are warranted to clarify the temporal dynamics of these alterations and their progression toward overt cardiometabolic disease.

This review has limitations that should be considered when interpreting the findings. The observational nature of the included studies precludes causal inference and leaves the possibility of residual confounding. In addition, between-study methodological and clinical heterogeneity was only partially accounted for at the meta-analytic level. Nevertheless, the review has several methodological strengths, including its comprehensive scope, the systematic assessment of risk of bias using the JBI tool, the parameter-specific interpretation of HRV findings according to their physiological meaning, and the application of meta-regression to explore sources of heterogeneity.

In conclusion, these findings suggest the presence of autonomic alterations in pediatric excess weight, while highlighting that their detectability depends strongly on the HRV metric examined. Short-term vagally mediated indices, particularly pNN50 and RMSSD, appear to be more sensitive to obesity-related autonomic impairment than global measures such as SDNN and MNN, whereas LF/HF may detect relevant alterations but requires especially cautious physiological interpretation. Overall, this parameter-dependent pattern reinforces the value of targeted HRV assessment as a clinically informative tool to identify early cardiometabolic risk and guide future preventive strategies in this population.

## Data Availability

The database that originated the article is available in an open repository: https://doi.org/10.5281/zenodo.20573253.
